# Therapeutic Effects of Baicalin on Diseases Related to Gut–Brain Axis Dysfunctions

**DOI:** 10.3390/molecules28186501

**Published:** 2023-09-07

**Authors:** Qichao Hu, Shuyu Hou, Baoyi Xiong, Yueqiang Wen, Jundong Wang, Jinhao Zeng, Xiao Ma, Fang Wang

**Affiliations:** 1 State Key Laboratory of Southwestern Chinese Medicine Resources, School of Pharmacy, Chengdu University of Traditional Chinese Medicine, Chengdu 611137, China; huqichaotcm@163.com (Q.H.); hsy2230311@163.com (S.H.); wjd@cdutcm.edu.cn (J.W.); 2 TCM Regulating Metabolic Diseases Key Laboratory of Sichuan Province, Hospital of Chengdu University of Traditional Chinese Medicine, Chengdu 610072, China; baoyixiong2002@163.com; 3 School of Clinical Medicine, Chengdu University of Traditional Chinese Medicine, Chengdu 610075, China; 4 School of Basic Medicine, Chengdu University of Traditional Chinese Medicine, Chengdu 611137, China; wenyueqiang@cdutcm.edu.cn; 5 Department of Gastroenterology, Hospital of Chengdu University of Traditional Chinese Medicine, Chengdu 610072, China; 6 Department of Pharmacy, Medical Supplies Center of PLA General of PLA General Hospital, Beijing 100039, China

**Keywords:** baicalin, the gut–brain axis, gut microbiome, Alzheimer’s disease, depression, obesity

## Abstract

The gut–brain axis is an active area of research. Several representative diseases, including central nervous system disorders (Alzheimer’s disease, Parkinson’s disease, and depression), metabolic disorders (obesity-related diseases), and intestinal disorders (inflammatory bowel disease and dysbiosis), are associated with the dysfunctional gut–brain axis. Baicalin, a bioactive flavonoid extracted from *Scutellaria baicalensis*, is reported to exert various pharmacological effects. This narrative review summarizes the molecular mechanisms and potential targets of baicalin in disorders of the gut–brain axis. Baicalin protects the central nervous system through anti-neuroinflammatory and anti-neuronal apoptotic effects, suppresses obesity through anti-inflammatory and antioxidant effects, and alleviates intestinal disorders through regulatory effects on intestinal microorganisms and short-chain fatty acid production. The bioactivities of baicalin are mediated through the gut–brain axis. This review comprehensively summarizes the regulatory role of baicalin in gut–brain axis disorders, laying a foundation for future research, although further confirmatory basic research is required.

## 1. Introduction

**Figure 1:**
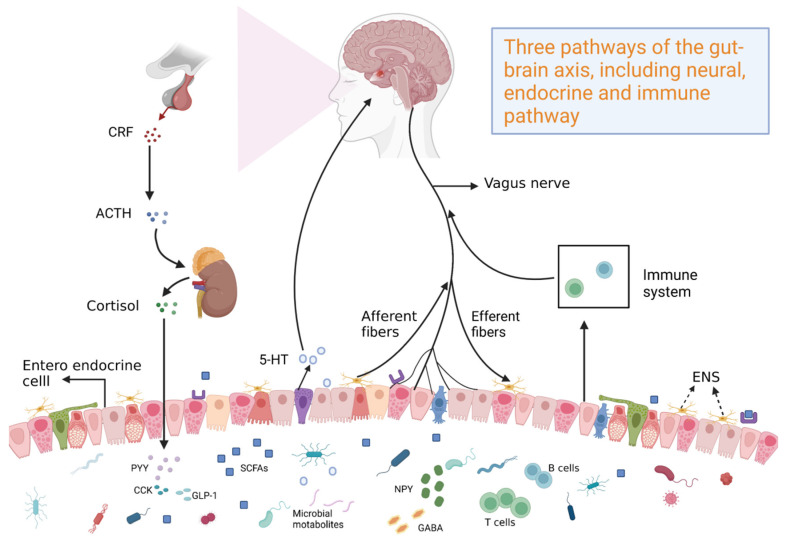
Profiles of bidirectional regulation of the gut–brain axis.

At the end of the twentieth century, research on neuroanatomy and the function and pathophysiology of gut–brain interaction rapidly progressed. Additionally, the field of neurogastroenterology and the concept of the gut–brain axis were established, which linked neuropsychiatry with gastroenterology. Theoretical understanding of the effects of the gut has markedly improved. The elucidation of the gut–brain axis revealed the bidirectional regulatory effects between the brain and the gut (Figure 1). In particular, the brain can project nerve impulses to affect intestinal microbiota and physiological function [1]. Conversely, intestinal microbiota can release metabolites, cytokines, and other signaling mediators to the central nervous system (CNS) through neuro-immune pathways, affecting the physiological function of the brain [2]. Currently, the gut–brain axis is suggested to be connected by the mutual activities of the CNS, the autonomic nervous system, and the enteric nervous system (ENS) [3]. The parasympathetic nerve is suggested to be a bridge connecting the CNS and the ENS. Thus, parasympathetic nerves, the CNS, and the ENS form the basic framework of the gut–brain axis [4,5]. However, previous studies have demonstrated the role of parasympathetic nerves in the regulation of the gut–brain axis [6,7]. The ENS is independent, as evidenced by its ability to regulate gastrointestinal functions without CNS innervation (e.g., gastrointestinal motility and secretion, intestinal blood flow, intestinal epithelial material transport, gastrointestinal immune responses, and regulation of inflammatory processes) [8]. Additionally, the ENS transmits gastrointestinal sensory signals out of the CNS and sympathetic ganglia through projection neurons [9]. The CNS occupies a dominant position, as evidenced by its regulatory effects on the ENS, mainly through gut microbiota, microbial products, and microbially produced metabolites in neuroinflammation and gastrointestinal diseases. The regulatory effect of the CNS on the gastrointestinal tract is mediated through the CNS-mediated integration of internal and external stimuli, which are transmitted to the enteric plexus through the nerve or neuroendocrine system or directly act on the gastrointestinal cells. Additionally, the hypothalamic–pituitary–adrenal (HPA) axis regulates the effects of stress on the gastrointestinal tract and gastrointestinal inflammation [10]. The gut–brain axis regulates gestational conditions, such as irritable bowel syndrome (IBS), inflammatory bowel disease (IBD), and gut microbiota composition. Among functional gastrointestinal diseases, IBS is the most prevalent disorder involving gut–brain interactions that are regulated by the gut–brain axis, which is related to intestinal motility and paresthesia, inflammation and immunity, and mental and psychological processes. These findings indicate the role of the gut–brain axis in the cross-talk between neurological disorders and intestinal disorders.

To understand the gut–brain axis, the brain and the gut must be considered a whole system that interacts. The brain and the gastrointestinal system mutually interact through crisscrossed main and collateral channels of the whole body. In particular, the physiological function of the brain depends on the nourishment of qi and blood produced by the gastrointestinal system [11]. Additionally, the emotional activities determined by the brain are related to the function of the intestine. For example, the syndrome of liver qi stagnation and spleen deficiency in traditional Chinese medicine (TCM) is caused by excessive emotional pressure, which leads to the physiological imbalance of spleen and stomach functions and symptoms, such as abdominal distension, pain, and diarrhea [12]. Although ancient Chinese medicine did not have a specific concept of the gut–brain axis, holistic treatments linked the brain and the intestine emerged and different TCM therapies were developed to alleviate relevant diseases based on the gut–brain axis. Among the various active natural products of TCM, several components modulate neurological disorders and intestinal disorders through the regulation of the gut–brain axis.

*Scutellaria baicalensis* is a perennial herb of the Lamiaceae family. The dried roots are often used as a traditional Chinese herbal medicine called Huangqin [13]. These roots, which have been popular since ancient times, contain several bioactive components, such as baicalin, baicalein, baicalein II, and wogonin [14,15]. In TCM, Huangqin is commonly used to stop diarrhea, lower blood pressure, relieve insomnia, and treat respiratory infections [16]. Baicalin, a bioactive flavonoid extracted from *Scutellaria baicalensis*, is a typical multi-target, multi-channel agent with multiple pharmacological effects, including hepatoprotective, antitumor, antibacterial, anti-inflammatory, antidepressant, and antioxidant effects [17,18]. For example, Chung et al. demonstrated that baicalin inhibits breast cancer by suppressing transforming growth factor (TGF)-β1-mediated epithelial–mesenchymal transition through the upregulation of the nuclear factor (NF)-κB pathway [19]. The value of natural products in the development and utilization of new drugs has piqued the interest of the scientific community. Baicalin has great research value owing to its wide range of pharmacological activities [20]. A recent study demonstrated that baicalin promotes the interaction of the liver–gut axis by regulating GPBAR1, farnesoid X receptor, bile acids, and microbiota [20]. This review aimed to elucidate the effects of baicalin on the gut–brain axis.

In this review, studies focusing on the therapeutic effects of baicalin on neurological and intestinal disorders mediated through the gut–brain axis were retrieved from the PubMed, Cochrane Library Web of Science, and EMBASE databases over the last five years. Studies that lacked scientific value or had obvious errors were excluded. All articles were used to systematically analyze the pharmacological effects of baicalin on the gut–brain axis, providing references for future studies.

## 2. Effects of Baicalin Functions on the Gut–Brain Axis

**Figure 2:**
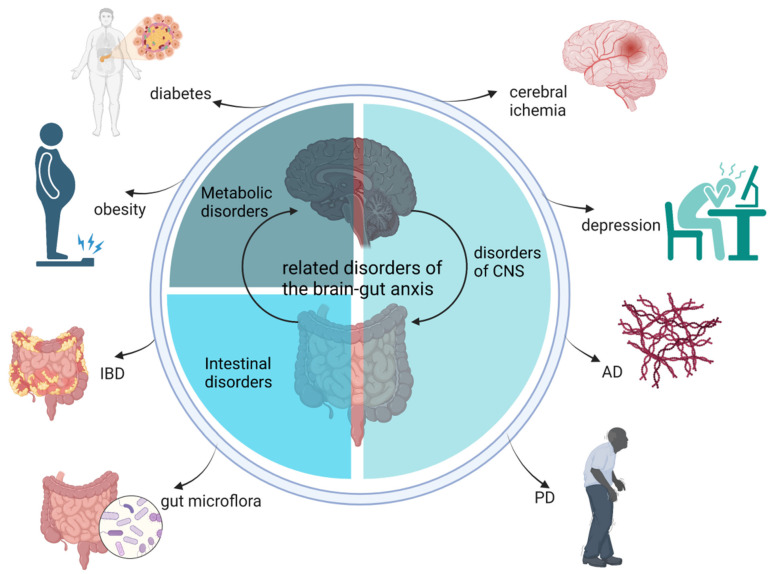
Related disorders in the gut–brain axis. AD, Alzheimer’s disease; PD, Parkinson’s disease; IBD, inflammatory bowel disease.

Several representative diseases, including CNS disorders (Alzheimer’s disease (AD), Parkinson’s disease (PD), and depression), metabolic disorders (obesity-related diseases), and intestinal disorders (IBD and regulation of the gut microbiota) are associated with the dysfunctional gut–brain axis (Figure 2).

### 2.1. Baicalin Alleviates CNS Disorders through the Gut–Brain Axis

The gut microbiota composition is dysregulated under stress conditions. This leads to the production of inflammatory mediators, such as IFN-γ, IL-1β, and IL-6, as well as the downregulation of short-chain fatty acids (SCFAs). These peripherally derived inflammatory factors may disrupt the blood–brain barrier (BBB) and migrate to the CNS, affecting the morphology of glial cells and microglia.

#### 2.1.1. Baicalin Mitigates Depression through the Gut–Brain Axis

A recent study reported that in the prefrontal cortex of chronically stressed rats, an increase in NF-κB, NLRP3, and IL-1β were upregulated in the prefrontal cortex of chronically stressed rats, which was accompanied by microglial activation and astrocyte injury [21]. Changes in these glial cells affect brain networks involved in memory, learning, mood, and emotion regulation, which can result in the onset of depression or anxiety disorders [22]. In developed countries, depression, a serious mental disorder, is considered to be one of the main causes of disability [23]. Depression is mainly characterized by low mood, anhedonia, irritability, concentration difficulties, and changes in appetite and sleep [24,25]. Previous studies have demonstrated that depression is related to impaired psychological health, cognitive impairment, increased risk of suicidal behavior, and increased mortality [26,27]. The pathophysiology of depression has not been completely elucidated. The monoaminergic deficiency hypothesis is the most commonly used hypothesis to explain depressive symptoms. According to this hypothesis, the levels of serotonin, norepinephrine, and dopamine are downregulated in the synaptic cleft of patients with depression [28]. Inflammatory processes, including the upregulation of pro-inflammatory cytokine levels and microglial activation, may influence behavior and mood [29]. Recent studies have reported that baicalin alleviates depression by inhibiting neuroinflammation. Guo et al. demonstrated that baicalin (60 mg/kg bodyweight) downregulated the levels of IL-1β, interleukin-6 (IL-6), and tumor necrosis factor-α (TNF-α) levels, decreased TLR4, and increased upregulated the phosphorylation of phosphatidylinositol 3-kinase (PI3K), Akt1, and Foxo1 in the hippocampus of the chronic unpredictable mild stress (CUMS)-induced depression mouse model. Treatment with 100 μM baicalin reversed damages in BV2 microglial cell lines. These results suggest that baicalin mitigates neuroinflammation-induced depression by downregulating TLR4 expression through the PIK3R1/AKT1/FOXO1 pathway [30]. Zhang et al. reported that baicalin (20 and 40 mg/kg bodyweight) suppressed CUMS-induced hippocampal apoptosis and inflammatory cytokines (Casp1 and IL-1β) and upregulated DCXcx, Eno2, and Bdnf in the hippocampus. These results suggest that baicalin exerts neuroprotective effects on the rat model of depression by inhibiting the Gsk3b/Rela/NLRP3 pathway [31]. Intestinal microbiota promotes NLRP3-mediated neuroinflammation in the hippocampus and mediates chronic ethanol exposure-induced depressive-like behavior [32]. Additionally, baicalin (20 and 40 mg/kg bodyweight) significantly mitigated the CUMS-induced activation of the NLRP3 inflammasome and upregulation of pro-inflammatory cytokines (IL-1β and IL-6) in the prefrontal cortex of rats, suggesting that baicalin exerts an antidepressant effect by inhibiting NLRP3inflammasome activation in the rat prefrontal cortex through the regulation of intestinal flora [33]. One study demonstrated that baicalin (60 mg/kg bodyweight) significantly promoted neuronal differentiation, increased the number of Dcx+ cells, and mitigated the CUMS-induced downregulation of p-Akt1, Foxg1, and Fgf2. These results suggest that baicalin exerts antidepressant effects by promoting neuronal differentiation through the Akt1/Foxg1 pathway [34]. Fang et al. reported that baicalin (50 mg/kg bodyweight) alleviated inflammatory pain-related depressive symptoms through Akt1-mediated adult hippocampal neurogenesis and promoted the growth and differentiation of hippocampal neural stem cells [35]. This indicated that baicalin mitigates depression and alleviates the altered behavior and hippocampal inflammation by targeting the Akt1 pathway through the modulation of gut microbiota composition and metabolites [36]. Li et al. established a mouse model of diarrhea-predominant IBS (IBS-D) using chronic restraint stress plus Senna Alexandrina Mill decoction. The IBS-D mouse model was then treated with baicalin (0.935 mg/d). The symptoms of depression, irritability, loss of appetite, and varying degrees of loose stools were alleviated in the experimental group. Baicalin downregulated the colonic levels of 5-hydroxytryptamine (5-HT) and Vip and the serum levels of 5-HT and Chat. Additionally, baicalin downregulated the colonic expression of NF-κBRela and regulated the level of basophil granulocytes and leukomonocytes in the whole blood, suggesting that baicalin alleviates inflammation. This study also indicated the critical role of baicalin in modulating the abundance of intestinal flora (*Bacteroidia*, Deferribacteres, Verrucomicrobia, Candidatus_Saccharibacteria, and Cyanobacteria) [37]. Baicalin treatment (20 and 40 mg/kg bodyweight) modulated Sirt1 and downregulated the level of Rela acetylation in the hippocampus and hypothalamus and the levels of pro-inflammatory cytokines (IL-1β, IL-6, and TNF-α) in mice with depression, suggesting that administration of baicalin may alleviate depression by modulating the Sirt1-NF-κB pathway [38]. SIRT1 is reported to be an important mediator of host-microbiome interactions and prevents intestinal inflammation by regulating the gut microbiota [39]. These findings suggest that baicalin exerts antidepressant effects by modulating the gut–brain axis, especially the gut microbes (Table 1).

#### 2.1.2. Baicalin Alleviates Cerebral Ischemia through the Gut–Brain Axis

Cerebral ischemia can damage the intestinal mucosal barrier and induce morphological damage in the intestinal mucosa, leading to intestinal dysbiosis and inflammation [60,61]. At month 12 post-cerebral ischemia, the serum levels of pro-inflammatory cytokines IFN-g, IL-6, and TNF-α are significantly upregulated. These pro-inflammatory cytokines from the intestinal tract can directly interact with each other, exacerbating pathological changes [62]. Cerebral ischemia, a global health issue caused by temporary or permanent, partial or total interruption of cerebral blood flow, is the second-leading cause of death and the third-leading cause of disability worldwide [63]. Based on the underlying neuropathology, stroke is classified as ischemic or hemorrhagic, with ischemic stroke accounting for 85% of all cases. Ischemic stroke is primarily caused by blockage of the middle cerebral artery, resulting in damage to the brain parenchyma in the affected area, followed by the induction of neuroinflammatory and immune responses [64]. Approximately 90% of stroke cases are reported to be related to behavioral factors, including malnutrition, low physical activity, and smoking, and metabolic factors, including diabetes, obesity, hyperlipidemia, and hypertension [65]. Brain damage from an ischemic stroke results from a complex series of neuropathological and neuropathological events, including excitotoxicity, oxidative stress, neuroinflammation, apoptosis, and amyloid production [66]. This section focuses on the correlation between the therapeutic effect of baicalin on cerebral ischemia and the brain–gut axis.

Liu et al. demonstrated that baicalin (50–100 mg/kg bodyweight) mitigated the cerebral ischemia and reperfusion-induced upregulation of trimethylamine (TMA) and TMA nitroxide and clusterin in middle cerebral artery occlusion (MCAO) mice. Additionally, treatment with baicalin (50 or 100 mg/kg bodyweight) significantly restored the level of intestinal flora, including the upregulation of the relative abundance of *Halomonas_smyrnensis* and the downregulation of the relative abundance of *Parabacteroides_johnsonii* and *Bacteroides_uniformis* in MCAO mice. The abundance of *Citromicrobium_sp_WPS32* and *Eubacterium_sp_CAG_86* was upregulated in MCAO mice treated with baicalin (100 mg/kg bodyweight), whereas that of *Lactobacillus_plantarum* was downregulated. Baicalin improved the cognition, memory, long-term potentiation, brain functional connectivity, and hippocampal neuronal plasticity of MACO mice. The intestinal flora of MACO mice was destroyed using broad-spectrum antibiotics. Treatment with antibiotics mitigated the neuroprotective effect of baicalin, which suggested that baicalin exerts neuroprotective effects in mice with cerebral ischemia-reperfusion injury by remodeling intestinal microorganisms [40]. Cerebral ischemia interrupts the transmission of GBA signals through the HPA axis [67]. Li demonstrated that baicalin (15 mg/kg bodyweight) mitigated the MCAO-induced neurological deficit and infarct volume by increasing the level of serum adrenocorticotropic hormone and the expression of the neuronal nucleus, glial fibrillary acidic protein, progesterone receptor, and serum progesterone in the ischemic penumbra in MCAO rats [41]. Hyperglycemia, which is an important risk factor for cerebral ischemia, promotes excessive activation of the HPA axis, leading to intestinal dysfunction [68,69]. Li et al. demonstrated that baicalin (100 mg/kg bodyweight) could regulate mitochondrial function through AMPK, alleviate hyperglycemia, and attenuate brain damage [42]. Although these two studies did not analyze GBA, we hypothesize that baicalin may regulate the HPA axis through different mechanisms and consequently affect the function of GBA and alleviate brain damage.

#### 2.1.3. Baicalin Alleviates AD through the Gut–Brain Axis

In AD, alterations in gut microbial composition promote the release of lipopolysaccharide (LPS) and amyloid protein [70]. Amyloid beta (Aβ) hyperactivates microglia, stimulating the NF-κB signaling pathway and promoting the release of pro-inflammatory cytokines, reactive oxygen species (ROS), and reactive nitrogen species, leading to neuronal and glial cell death [71]. AD, a progressive multifactorial neurodegenerative disease, is characterized by severe deficits in memory, cognition, and motor functions, which result in decreased mental, behavioral, and functional activities and poor quality of life [72]. The total number of people living with AD is projected to reach 139 million in 2050, making it a global health challenge [73].

Cognitive dysfunction may be due to the deposition of extraneuronal Aβ plaques and intraneuronal tau tangles, leading to neuroinflammation, altered calcium homeostasis, vascular degeneration, and ultimately neuronal death [74]. Dietary flavonoids regulate the NF-κB signaling pathway, which is a potential therapeutic target for AD. Flavonoids, a subtype of polyphenols, modulate microbiota-related metabolism, improve neurological health, activate the anti-apoptotic survival signaling pathways, upregulate antioxidant gene expression, and alleviate Aβ pathology, exerting therapeutic effects on neuronal dysfunction and toxicity. Flavonoids and other sugars that are not absorbed in the small intestine are broken down by the intestinal microbiota into phenolic acids and other metabolites (such as galactose and uronic acid), which inhibit the growth of *Ruminococcus gaunii*, *Bacteroides*, and *Lactobacillus*. Harach et al. reported that the number of *Bacteroidetes* was downregulated in the intestine of APPS1 mice, while Aβ-induced pathology was alleviated in the brain of sterile amyloid precursor protein (APP) transgenic mice [75], which suggested that changes in the number of microbial strains may lead to amyloid protein deposition, triggering an immune response and consequently leading to AD. Additionally, flavonoids play an important role in the prevention of neuritis as evidenced by the inhibition of inflammatory cytokine (TNF-α and IL-1β) production in microglia and Nos2 and ROS production in glial cells and the downregulation of pro-inflammatory transcription factors, such as NF-κB, which are mediated through the regulation of the glial and neuronal signaling pathways [76]. Baicalin, a herbal flavonoid, was demonstrated to exert these effects in several studies. Jin et al. reported that baicalin (100 mg/kg bodyweight) alleviates spatial memory impairment in Aβ precursor protein/presenilin-1 (PS1) mice and effectively reduces the levels of pro-inflammatory factors (IL-6, TNF-α, IL-1β) and neuroinflammation-mediated neuronal apoptosis. These results suggested that baicalin can inhibit microglia-induced neuroinflammation, alleviate cognitive impairment, and protect neurons by inhibiting the activation of NLRP3 inflammasome and TLR4/NF-κB signaling pathway [43]. The accumulation and deposition of Aβ in senile plaques and cerebral blood vessels may contribute to the progressive neurodegeneration in AD. The oral administration of baicalin (30, 50, and 100 mg/kg bodyweight) attenuated Aβ1–42 protein-induced glial cell activation and upregulation of TNF-α and IL-6, suggesting that baicalin ameliorates Aβ1–42 protein-related pathology and cognitive dysfunction by exerting anti-inflammatory effects. Thus, baicalin is a potential therapeutic drug for AD [44]. Similarly, baicalin (50, 100, and 200 mg/kg bodyweight) treatment restored the activity of antioxidant enzymes (superoxide dismutase (SOD), catalase (CAT), and glutathione peroxidase (GPx)) and upregulated their gene expression, improving antioxidant capacity, inhibiting Bax/Bcl2 expression, cytochrome c release, and CASP9/CASP3 activation. Baicalin can effectively improve Aβ-induced learning and memory impairment, hippocampal damage, and neuronal apoptosis [45]. Additionally, baicalin (50 and 100 μM) inhibited the β-amyloid peptide (Aβ42)-induced proliferation of BV2 microglial cells, downregulated the expression of CD11b, decreased the chemotactic ability of BV2 cells, significantly inhibited the production of IL-6, TNF-α and NO, and suppressed Aβ-induced phosphorylation of Jak2 and STAT3, suggesting that baicalin can prevent Aβ-induced microglial activation by inhibiting the JAK2/STAT3 signaling pathway [46].

#### 2.1.4. Baicalin Alleviates PD through the Gut–Brain Axis

In PD, gut dysbiosis stimulates the production of inflammatory cytokines and LPS, leading to intestinal epithelial damage and impaired barrier integrity [77]. Increased intestinal permeability leads to microbial translocation and the introduction of bacterial toxins and host-derived inflammatory cytokines (TNF-α, IL-6, and IL-10) into the bloodstream, directly contributing to the nervous system impairment owing to the disruption of the BBB integrity. The interaction provides a pathway [78], and these cytokines are significantly elevated in the serum of patients with PD, suggesting that the gut–brain axis may be associated with symptom severity and disease progression in PD [79].

**Figure 3:**
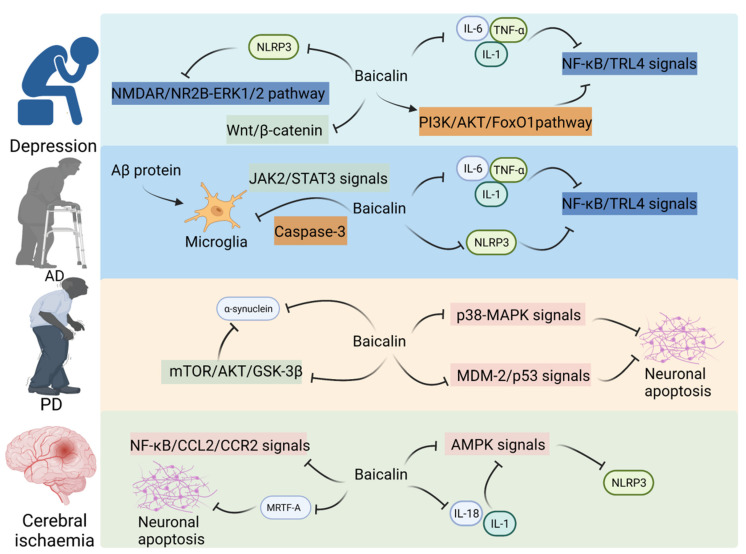
Baicalin on CNS disorders in the gut–brain axis.

Dodiya et al. demonstrated that low-dose oral rotenone-induced intestinal hyper-permeability and/or microbial dysbiosis leads to pro-inflammatory intestinal milieu, aggravating PD phenotype in the PD mouse model, suggesting that the microbiota–gut–brain axis is involved in the pathogenesis of PD [80]. Lei et al. reported that baicalin (20 and 40 mg/kg bodyweight) protected dopaminergic (DA) neurons against ROS-induced damage and downregulated the expression of Cebpb and Snca, and alleviated the 1-methyl-4-phenyl-1,2,3,6-tetrahydropyridine (MPTP)-induced loss of substantia nigra DA neurons in PD mice. These results suggest that baicalin exerts therapeutic effects on PD by inhibiting the expression of Cebpb [47]. One study revealed that baicalin (25 mg/kg bodyweight) significantly mitigated 6-hydroxydopamine (6-OHDA)-induced neuronal apoptosis in PD rats and downregulated the expression of Snca, Mtor, Akt1, and Gsk3b in rats. These findings suggest that baicalin may inhibit the apoptosis of substantia nigra neurons in PD rats by suppressing the mTOR/Akt1/GSK-3β pathway [48]. Huang et al. revealed that the concentrations of some SCFAs, especially propionate, were significantly downregulated in the stool samples of patients with PD. Propionate administration significantly improved intestinal epithelial barrier function and motor behavior in the MPTP-induced PD mouse model through the Akt1 signaling pathway [81]. Additionally, baicalin mitigated 6-OHDA-induced cell damage and exerted therapeutic effects on PD by downregulating miR-192-5p and suppressing the PI3K/Akt1 and Mdm2/Tp53 signaling pathways [82] (Figure 3).

### 2.2. Therapeutic Effects of Baicalin on Metabolic Disorders

The intestinal microbiome plays an important role in the metabolism of undigested nutrients, fiber, and bile acid in the diet entering the large intestine [83,84]. The intestinal microbiota metabolizes these nutrients to release metabolites, such as SCFA, indole, and secondary bile acids. These metabolites interact with intestinal cells, promoting hormone secretion and regulating the balance of appetite homeostasis in the brain. Intestinal inflammation affects cells in the gut and gut microbiota, promoting the production and release of metabolites that act on the CNS. The disruption of gut microbiota can promote inflammatory reactions and exacerbate human metabolism, leading to metabolic disorders, such as non-alcoholic fatty liver disease and hyperlipidemia [28,85]. These results suggest that the dysregulation of the gut–brain axis promotes the development of diabetes, obesity, and other related metabolic disorders.

Hormones are involved in the regulation of food intake homeostasis. For example, gut hormones that promote appetite and anorexia, leptin signaling in adipose tissue and dopamine reward systems, and prefrontal suppression are closely related. These peripheral signals partially act through specific hypothalamic nuclei. Gut microbiota regulates the release of endocrine hormones in the gut through metabolism, altering the balance of appetite signals in the hypothalamus [86]. In the gastrointestinal tract, vagus nerve sensory neurons regulate gastric volume and intestinal contents. LGLE+ vagus nerve, especially the Glp1r+-type in the gastric myenteric layer, may be the first to transmit the gastric dilatation signal after eating to the brain stem [87]. Next, the ingested nutrients activate the intestinal endocrine cells, which respond to nutrients by releasing a large number of intestinal hormones, including glucagon-like peptide-1 (GLP-1), cholecystokinin (CCK), glucose-dependent insulin-stimulating peptide (GIP), and incretin, which can act on the CNS through the vagus nerve [88]. Additionally, these hormones regulate glucose metabolism and fat metabolism by acting on metabolism-related cells. For example, glucose-dependent insulinotropic peptides can act on pancreatic beta cells and adipocytes, promote adipocyte fat storage, and directly induce satiety through the hypothalamus [89]. GLP-1 can directly act on islet beta cells to accelerate insulin secretion and reduce glucagon secretion, lowering blood sugar [90].

Obesity has traditionally been defined as excess body fat leading to impaired health and is usually assessed in clinical practice based on body mass index [91]. The biology of obesity and energy balance has been extensively studied. However, the current understanding has not markedly delayed the obesity epidemic. Obesity, which is a global health burden, is a modifiable risk factor for cardiovascular disease and is not adequately addressed by lifestyle changes or pharmacotherapy when compared with hypertension, dyslipidemia, diabetes, and smoking [92]. In this section, the correlation between the therapeutic role of baicalin in metabolic disorders and the brain–gut axis has been examined (Figure 3).

Ju et al. demonstrated that baicalin effectively regulated the blood glucose metabolism of high-fat diet (HFD)-fed mice, including the downregulation of fasting blood glucose and the upregulation of postprandial blood glucose metabolism, suppressing fat accumulation and liver injury caused by long-term HFD consumption and alleviating dysregulated blood lipid metabolism. Baicalin significantly mitigated the HFD-induced downregulation of *Ackermann bacteria*, cocci, and rumen cocci abundances and the upregulation of odorous bacteria and Parabacillus abundances. The abundance of these bacteria in baicalin-treated HFD mice was similar to those in healthy control mice [58]. *Ackermann bacteria*, *Ruminococcus*, and *Pseudomonas* mainly produce acetic acid. Staphylococcus mainly produces propionic acid and butyric acid [93]. These SCFAs promote the metabolism, growth, and differentiation of intestinal cells, protect the intestinal mucosa, and alleviate local inflammatory reactions [94]. SCFAs interact with intestinal cells to produce GLP-1, CCK, GIP, and incretin. These hormones may act on the CNS through the vagus nerve and interact with specific receptors in the brain to modulate food intake and appetite. These results indicate that baicalin may modulate metabolic disorders by regulating the CNS through the modulation of intestinal microbiota. Lin et al. reported that 8 weeks of treatment with baicalin (400 mg/kg bodyweight) alleviated liver fat accumulation, dysregulated blood lipids, weight loss, and insulin sensitivity in the HFD-induced metabolic syndrome (MetS) mouse model through the regulation of citric acid cycle (tricarboxylic acid (TCA) cycle), alanine, aspartic acid, and glutamic acid metabolism, and glycerol phospholipid metabolism. Baicalin changed the composition and function of intestinal microbiota in MetS mice by regulating the TCA cycle and aminoacyl tRNA biosynthesis. In particular, baicalin exerts protective effects on the intestine by regulating the TCA cycle. Baicalin prevented the decline of the relative abundance of Bacteroides and the proportion of Bacteroides and Firmicutes in HFD-fed mice. Additionally, the change in succinic acid, an important product of the TCA cycle, was negatively correlated with the abundances of Bacteroides and Sutterella and positively correlated with the abundances of *Myxosporidium*. Treatment with succinic acid for 8 weeks significantly decreased the bodyweight and low-density lipoprotein-cholesterol levels of MetS mice. Hematoxylin and eosin revealed that baicalin downregulated fat accumulation. These results indicate that succinic acid exerts protective effects in MetS mice. Treatment with antibiotics to destroy the intestinal flora suppresses the protective effects of baicalin, indicating that baicalin exerts protective effects through the intestinal flora [49]. Although these two studies did not examine the CNS of mice, the effect of baicalin on the intestinal tract was apparent. The effects of many metabolites generated from the intestinal tract on the CNS are clear. Therefore, we predicted that baicalin regulates intestinal flora, modulates the CNS, and consequently exerts therapeutic effects on metabolic disorders.

### 2.3. Baicalin Alleviates Intestinal Disorders

Disturbances in the gut are correlated with metabolic disorders. Several studies have reported the correlation between IBD, neurodegenerative, and neuroinflammatory diseases [95,96]. Recent population-based studies suggest that patients with IBD are at a high risk of developing PD [97]. The vagus nerve exerts anti-inflammatory effects by inhibiting the cholinergic efferent fibers that release inflammatory cytokines from macrophages [98]. Thus, vagal nerve stimulation downregulates intestinal immune cells and clinical markers of inflammation in IBD [99]. In IBS, vagus nerve stimulation can alleviate constipation and abdominal pain and downregulate circulating IL-6 and TNF-α levels [100].

#### 2.3.1. Baicalin Alleviates IBD through the Gut–Brain Axis

IBDs, a global healthcare problem with increasing incidence [101], including Crohn’s disease and ulcerative colitis (UC), are characterized by chronic relapse of intestinal inflammation [102]. Previous studies have suggested that IBD is caused by aberrant persistent immune responses against gut microbes that can be attributed to the genetic predisposition of individuals [103]. Although the etiology of IBD is largely unknown, it involves a complex interplay between genetic, environmental, or microbial factors and immune responses [104]. This section mainly discusses the correlation between the therapeutic effect of baicalin on UC and the brain–gut axis.

Zhu et al. demonstrated that baicalin (50 μM) suppressed LPS-induced M1 macrophage polarization and upregulation of TNF- α, IL-23, and interferon regulatory factor 5 (IRF5) in vitro, as well as upregulated the expression of IL10, ARG1, and IRF4. These factors alleviate intestinal inflammation. In vivo experiments revealed that baicalin (100 mg/kg bodyweight) inhibited the expression of IRF5 protein, promoted the expression of IRF4 protein, induced M2-type polarization of macrophages, and improved the disease activity index of dextran sodium sulfate (DSS)-induced colitis in mice [50,105]. The microglial expression of IRF5 and IRF4 was related to pro-inflammatory and anti-inflammatory responses, respectively, which contributed to the progression of poststroke inflammation and led to secondary neuronal damage. Additionally, the IRF5–IRF4 regulatory axis was suggested to be a crucial factor in microglial activation and a potential therapeutic target for neuroinflammation and ischemic stroke [106]. Baicalin (60 and 90 mg/kg bodyweight) can significantly increase the activities of SOD, CAT, and GPx in the colon tissue of UC rats and significantly downregulate the levels of malonaldehyde (MDA), IL-1β, myeloperoxidase, cleaved Casp3, cleaved Casp9, Bcl2/Bax, Cycs, and PEG2in the colon tissue of UC rats. These results suggest that baicalin exerts therapeutic effects on UC by inhibiting the IKK/IKB/NF-κB signaling pathway and apoptosis-related proteins [51]. However, Magdy et al. indicated that the NF-κB pathway alleviated glutamate accumulation and neuronal cell damage by regulating the expression of membrane proteins (connexins (Cxs), Panx1, and excitatory amino-acid transporters) in the brain tissue [107]. Baicalin (25, 50, and 100 mg/kg bodyweight) protected against trinitrobenzenesulfonic acid (TNBS)-induced UC in rats by inhibiting the upregulation of ROS and MDA in the colon tissue of rats, downregulation of the GSH and SOD levels, and modulation of Th17/T regulatory (Treg) cell balance, gut microbiota, and SCFAs [52]. These results suggest that baicalin alleviates UC-related inflammation and intestinal dysbiosis. Baicalin (316 µg/mL) significantly inhibited the release of TNF-α, IL-6, and IL-1β, promoted the expression of IL-10, and significantly suppressed LPS-induced apoptosis by blocking the PI3K/AKT signaling pathway [53]. Additionally, baicalin (50, 100, and 150 mg/kg bodyweight) downregulated the release of TNF-α, IL-1β, and IL-6 in the serum of DSS-induced chronic UC mice and suppressed IL-33 and NF-κB [54]. Similarly, treatment with baicalin (10 mg/kg bodyweight) significantly downregulated the levels of inflammatory mediators, including MPO activity, TNF-α, IL-1β, and Th1-related cytokines (IL-12 and IFN- c), decreased the number of Th17 cells, and suppressed the levels of Th17-related cytokines (IL-17 and IL-6) [55]. Similarly, baicalin can improve the inflammatory symptoms of induced colitis, downregulate the levels of pro-inflammatory mediators (TNF-α and IL-1β) in the colonic mucosa, and suppress the expression of Rela protein [56]. Additionally, baicalin can simultaneously downregulate the expression of migration inhibitory factors, including CCL2 and CCL20, and the number of macrophages in UC rats [57].

#### 2.3.2. Baicalin Modulates Gut Microbes

Recent studies have demonstrated that gut microbiota, including bacteria, fungi, protozoa, and viruses, mediate gut–brain signaling. This has led to the emergence of the concept of a microbe–gut–brain axis [108]. The gut microbiota comprises trillions of microbes from thousands of species with 100 times more unique microbial genes than human genes. These microbes, which produce various metabolites that regulate other bacteria, are localized in the intestinal epithelium, bind to protein targets, and modulate the host functions through the intestinal barrier [109,110].

In the HFD-induced MetS mouse model, baicalin (200 mg/kg bodyweight/day) upregulated the number of SCFA-producing bacteria in the intestine, enhanced the metabolism of the intestinal cells, and alleviated MetS in mice. Additionally, hypertension can cause intestinal barrier damage [58]. For example, previous studies have investigated the therapeutic effect of baicalin (100 mg/kg bodyweight) on intestinal barrier injury in spontaneously hypertensive rats. The experimental outcomes indicated that baicalin administration increased the number of SCFA-producing bacteria and changed the intestinal microecosystem, leading to the alleviation of hypertension-induced intestinal barrier damage [59]. Zhu et al. demonstrated that baicalin (25–100 mg/kg bodyweight) exerted effective therapeutic effects on TNBS-induced UC by upregulating the number of SCFA-producing bacteria, especially butyrate-producing bacteria, such as *Butyricimonas* spp., *Roseburia* spp., *Subdoligranulum* spp., and *Eubacterium* spp. [52].

## 3. Discussion and Outlook

The discovery of the gut–brain axis has provided a new avenue to understand the pathogenesis of diseases. The disorders of the gut–brain axis can be mainly divided into CNS, metabolic, and intestinal disorders. Various studies have demonstrated that baicalin regulates the gut–brain axis. This narrative review provides a new perspective for understanding the therapeutic potential of baicalin in the gut–brain axis by comprehensively discussing the therapeutic role of baicalin in the disorders of the gut–brain axis.

**Figure 4:**
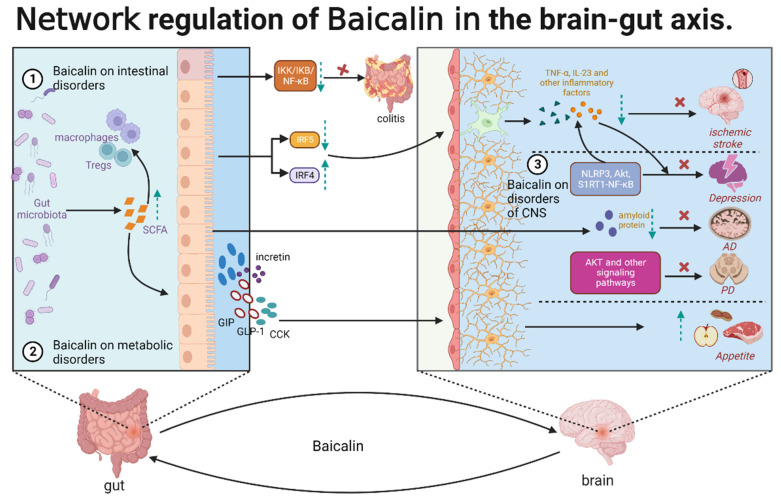
The role of baicalin in the gut–brain axis.

Baicalin exerts therapeutic effects on the gut–brain axis through the regulation of CNS, metabolic, and intestinal disorders. Additionally, baicalin exhibits multi-dimensional activities at the systemic, cellular, and molecular levels, forming a complex network relationship, which is the basis for the therapeutic effects of baicalin on various cerebral and intestinal diseases related to the gut–brain axis. In particular, baicalin indirectly regulates the components of refractory diseases that are inaccessible due to some physiological structures, such as BBB and intestinal wall barrier. In CNS disorders, baicalin exerts anti-neuroinflammatory and anti-neuronal apoptotic effects. Baicalin inhibits microglia activation by regulating the NLRP3, AKT1, and S1RT1-NF-κB signaling pathways and consequently alleviates neuroinflammation and depression. Additionally, baicalin can alleviate AD by regulating intestinal microbiota and decreasing the deposition of amyloid protein. Furthermore, baicalin inhibited neuronal apoptosis through AKT and other signaling pathways and alleviated PD and cerebral ischemia. In metabolic disorders, baicalin can balance intestinal flora composition, increase the production of SCFAs, and interact with intestinal cells to induce the production of GLP-1, CCK, GIP, and incretin. These hormones may act on the CNS through the vagus nerve to alter food intake and appetite. In intestinal diseases, the therapeutic effects of baicalin are multifaceted. Baicalin induces the gut microbiota to release SCFAs, which are involved in inflammation regulation by interacting with intestinal immune cells, such as macrophages and dendritic cells. Additionally, baicalin directly participates in intestinal inflammatory responses by inhibiting the IKK/IKB/NF-κB signaling pathway. Furthermore, baicalin may inhibit the activation of microglia through the IRF5–IRF4 regulatory axis, alleviating neuroinflammation and cerebral ischemia. These findings indicate that baicalin is involved in the regulation of the CNS, gut, and metabolism network through the gut–brain axis. This regulation may be mediated by gut microbes, glial cells, or gut immune cells (Figure 4). Intestinal flora can remove the glycoside on the 7-hydroxyl group of baicalin, resulting in the formation of baicalein. Thus, baicalin can exert therapeutic effects on intestinal diseases through its metabolites. Recent studies suggested a link between aberrant gut microbiota and post-traumatic stress disorder (PTSD) and indicated baicalin is a potential therapeutic target for PTSD [111]. Baicalein, a well-known precursor compound of baicalin, alleviated PTSD-induced anxiety and pain by modulating the serotonergic system and spinal δ-opioid receptors [112]. However, low bioavailability limits the clinical application of baicalin. New dosage forms or structural modifications, such as baicalin methyl ester and baicalin-7-methyl ether, must be developed to improve the pharmacokinetic parameters of baicalin and promote its clinical application.

Most studies included in this review focused on the therapeutic effect of baicalin on specific disease models. However, the therapeutic effects of baicalin have not been evaluated using related cellular studies. Microglia, astrocytes, macrophages, and dendritic cells play important regulatory roles in gut–brain axis disorders. Additionally, gut–brain axis disorders are closely related to other disorders, such as substance use disorders [113]. The gut microbiota and its secreted components, such as SCFAs, can further regulate CNS disorders. Therefore, the role of baicalin in the gut–brain axis may be a breakthrough finding.

Although several experimental studies have examined the pharmacological effects of baicalin on the gut–brain axis, limited numbers of clinical studies on baicalin have been performed. Thus, the clinical application of baicalin for the treatment of diseases may be delayed. This review has several limitations. For example, the therapeutic efficacy of baicalin may vary in different disease models. Additionally, key factors, such as clinical dose, dosage form, and mode of administration, varied between the studies. Therefore, baicalin must be comprehensively analyzed in different disease models before application in clinical trials to determine the most suitable model.

## 4. Conclusions

This review comprehensively discussed the therapeutic potential of baicalin in the gut–brain axis and elucidated its roles in CNS, metabolic, and intestinal disorders. Further studies are required to achieve clinical translation of baicalin. However, available evidence indicates the therapeutic value of baicalin in the disorders of the gut–brain axis.

## Figures and Tables

**Table 1:** Mechanisms of baicalin in gut–brain axis related disorders.

Category	Related Disorders	Animal/Cell Model	Dose	Target Pathway/Mechanisms	Reference
Disorders in CNS	Depression	chronic unpredictable mild stress (CUMS) mice/BV2 microglia cell lines	60 mg/kg (in vivo); 100 μM (in vitro)	↓ TLR4; ↓ PI3K/AKT/FoxO1 signaling pathway	[30]
		CUMS mice	20 and 40 mg/kg (in vivo)	↓ GSK3β/NF-κB/NLRP3 signaling pathway	[31]
		CUMS mice	20 and 40 mg/kg (in vivo)	↓ NLRP3 signaling pathway	[33]
		CUMS mice	60 mg/kg (in vivo)	↓ Akt/FOXG1 signaling pathway	[34]
		CUMS mice	50 mg/kg (in vivo)	↓ Akt signaling pathway	[35]
		CUMS mice	20 and 40 mg/kg (in vivo)	↓ SIRT1-NF-κB signaling pathway	[38]
	Cerebral ischaemia	MCAO (middle cerebral arteryocclusion) rats	100 mg/kg (in vivo)	/	[39]
		MCAO rats	50–100 mg/kg (in vivo)	remodeling the gut microbiota	[40]
		MCAO rats	15 mg/kg (in vivo)	↑ ACTH; ↑ NeuN; ↑ GFAP	[41]
		MCAO rats	100 mg/kg (in vivo)	↓ AMPK signaling pathway	[42]
	Alzheimer’s disease	APP (amyloid beta precursor protein)/PS1 (presenilin-1)mice	100 mg/kg (in vivo)	↓ NLRP3 signaling pathway; ↓ TLR4/NF-κB signaling pathway	[43]
		amyloid β (Aβ)_1–42_ protein-induced AD mouse	30, 50 and 100 mg/kg (in vivo)	↓ Aβ_1–42_ protein	[44]
		amyloid β (Aβ)_1–43_ protein-induced AD mouse	50, 100 and 200 mg/kg (in vivo)	↓ Aβ_1–43_ protein; ↓ Caspase-3 signaling pathway	[45]
		amyloid β (Aβ)_1–44_ protein-induced BV2 microglial cells	50 and 100 μM (in vitro)	↓ JAK2/STAT3 signaling pathway	[46]
	Parkinson’s disease	1-methyl-4-phenyl-1,2,3,6-tetrahydropyridine (MPTP) induced PD mouse	20 and 40 mg/kg (in vivo)	↓ C/EBPβ	[47]
		6-hydroxydopamine (6-OHDA)-induced PD rats	25 mg/kg (in vivo)	↓ mTOR/AKT/GSK-3β signaling pathway	[48]
Metabolic disorders	Obesity	HFD-induced obesity mice	400 mg/kg/day (in vivo)	/	[49]
Disorders in gut	IBD	DDS-induced UC	25, 50 and 100 mg/kg (in vivo); 6.25, 12.5, 25, and 50 μM (in vitro)	↓ IRF5; ↑ IRF4; ↓ TNF-α/IL-23/IRF5; ↑ IL-10/Arg1/IRF4	[50]
		TNBS-induced UC	30–90 mg/kg (in vivo)	↓ cleaved-caspase3/cleaved-caspase9/Bcl-2/Bax/cyt-c/NF-kB p-65/p-IKKβ/IKKβ and p-IKBα/IKBα ↓ p-IKBα/IKBα ↓ MDA/PEG2/MPO/IL-1β/TNF-α	[51]
		TNBS-induced UC	25, 50 and 100 mg/kg (in vivo)	↓ Th17/Treg signaling pathway	[52]
		TNBS-induced UC	100 mg/kg/d (in vivo); 316 µg/mL (in vitro)	↓ PI3K/AKT signaling pathway	[53]
		DDS-induced UC	50, 100 and 150 mg/kg (in vivo)	↓ MPO,NO,Ly6/G,IL-6,IL-1β,TNF-α ↓ IL-33/NF-κB p65/p-NF-κB p65/p-IκB-α; ↑ IκB-α	[54]
		TNBS-induced UC	10 mL/kg (in vivo)	↓ MPO/TNF-α/IL-1β/IFN-γ/IL-12; ↓ Th17/Treg	[55]
		TNBS-induced UC	25, 50 and100 mg/kg (in vivo); 5 × 10^−4^, 5 × 10^−5^, 5 × 10^−6^ μM (in vitro)	↓ TLR4/NF-κB signaling pathway;	[56]
		TNBS-induced UC	10 mL/kg (in vivo)	↓ MIF/MCP-1/CCL2/MIP-3α/CCL20	[57]
	Microbiota regulation	high-fat diet-induced disorder	200 mg/kg (in vivo)	SCFAs	[58]
		TNBS-induced disorder	20–100 mg/kg (in vivo)	SCFAs	[52]
		intestinal barrier damage-induced disorder	100 mg/kg (in vivo)	SCFAs	[59]

↑: up-regulation; ↓: down-regulation.

## Data Availability

All data are available in the manuscript, and they are shown in the figures and tables.

## Abbreviations

EMT: epithelial–mesenchymal transition; FXR: farnesoid X receptors; CNS: central nervous system; ENS: enteric nervous system; HPA: hypothalamic–pituitary–adrenal; IBS: irritable bowel syndrome; SCFAs: short-chain aliphatic hydrocarbons; BBB: blood–brain barrier; AD: Alzheimer’s disease; TCM: traditional Chinese medicine; LPS: lipopolysaccharide; PD: Parkinson’s disease; GLP-1: glucagon-like peptide-1; CCK: cholecystokinin; GIP: glucose-dependent insulinotropic peptide; IBD: inflammatory bowel disease; IBS: irritable bowel syndrome; IL-1β: interleukin-1β; IL-6: interleukin-6; TNF-α: tumor necrosis factor-α; PI3K: phosphatidylinositol 3-kinase; AKT: protein kinase B; FoxO1: factor forkhead box protein O 1; NAFLD: non-alcoholic fatty liver disease; NASH: non-alcoholic steatohepatitis; CUMS: chronic unpredictable mild stress; CMS: chronic mild stress; NLRP3: nucleotide-binding domain; NSE: neuron-specific enolase; BDNF: brain-derived neurotrophic factor; CEE: chronic ethanol exposure; DCX+: doublecortin; SIRT1: Sirtuin 1; GSK3β: glycogen synthase kinase-3; CREB: element-binding protein; MCAO: middle cerebral artery occlusion; TMA: trimethylamine; TMAO: trimethylamine nitroxide; NeuN: neuronal nuclei; GFAP: glial fibrillary acidic protein; ACTH: adrenocorticotropic hormone; MRTF-A: myocardin-related transcription factor-A; PS1: precursor protein/presenilin-1; Aβ: accumulation and deposition of beta-amyloid peptide; Aβ42: β-amyloid peptide; α-syn: α-synuclein; ROS: reactive oxygen species; BMI: body mass index; BAT: brown adipose tissue; WAT: white adipose tissue; UCP1: coupling protein 1; iBAT: interscapular brown adipose tissue; ATM: adipose tissue macrophages; IBD: inflammatory bowel disease; UC: ulcerative colitis; IRF5: interferon regulatory factor 5; LPS: lipopolysaccharide; MIF: macrophage migration inhibitory factor; BASO: basophil granulocyte; LYMPH: leukomonocyte; NF-κB: nuclear factor kappa-B; IBS-D: diarrhea-predominant irritable bowel syndrome; VIP: vasoactive intestinal polypeptide; CHAT: choline acety transferase; PTSD: post-traumatic stress disorder.
